# Validation of Insole Pressure Sensor Algorithms: Implications for In-Field Detection of Initial Contact and Hamstring Muscle Pre-Activity During Side-Cutting

**DOI:** 10.3390/s26113539

**Published:** 2026-06-03

**Authors:** Emilie E. Zwicky, Niels J. Nedergaard, Tine Alkjær, Connie Linnebjerg, Mathias M. Nikolajsen, Hanne B. Lauridsen, Mette K. Zebis

**Affiliations:** 1 Team Danmark, The Elite Sport Organization of Denmark, 2605 Brondby, Denmark; emzw@teamdanmark.dk (E.E.Z.); hbla@teamdanmark.dk (H.B.L.); 2 Department of Midwifery, Physiotherapy, Occupational Therapy and Psychomotor Therapy Faculty of Health, University College Copenhagen, 2200 Copenhagen N, Denmark; njen@kp.dk (N.J.N.); clin@kp.dk (C.L.); 3 Department of Biomedical Sciences, University of Copenhagen, 2200 Copenhagen N, Denmark; talkjaer@sund.ku.dk (T.A.); mathias.nikolajsen@sund.ku.dk (M.M.N.); 4 The Parker Institute, Bispebjerg and Frederiksberg Hospital, 2000 Frederiksberg, Denmark; 5 Institute of Sports Medicine Copenhagen, Department of Orthopedic Surgery, Copenhagen University Hospital Bispebjerg and Frederiksberg, 2400 Copenhagen NV, Denmark

**Keywords:** plantar pressure insoles, wearable sensors, initial contact, medial hamstring, change-of-direction, ACL injury

## Abstract

**Highlights:**

**What are the main findings?**

- A purpose-built criteria-based insole pressure sensor algorithm reduced systematic bias in initial contact detection during sport-specific side-cutting, thereby enabling valid electromyographic measures of m. semitendinosus pre-activity.
- The commonly used default setup—body weight-threshold initial contact detection using insole pressure sensors—systematically delays initial contact identification and consequently underestimates m. semitendinosus pre-activity during side-cutting.

**What are the implications of the main findings?**

- The combination of insole pressure sensors with the criteria-based initial contact detection algorithm, and surface electromyography shows potential for assessing semitendinosus pre-activity during side-cutting maneuvers outside laboratory environments.
- This approach may facilitate large-scale in-field monitoring of hamstring muscle activity during side-cutting maneuver, supporting research on ACL injury risk assessment, and prevention program evaluation.

**Abstract:**

Accurate detection of initial contact (IC) during side-cutting is essential for evaluating m. semitendinosus (ST) pre-activity, a protective mechanism against ACL injury in team sport athletes. This study developed two insole pressure sensor (IPS) algorithms—a body weight-based and a criteria-based algorithm—for IC detection and evaluated their agreement with force-plate-derived IC based on vertical ground reaction forces (vGRF). Twenty-six adult female athletes performed sport-specific side-cutting while IPS, vGRF, and ST electromyography were recorded. IPS-derived IC events were compared with vGRF-derived IC, and ST pre-activity within 50 ms prior to IC was compared between methods. Agreement and limits of agreement (LoA) were evaluated using Bland–Altman analysis. The body weight-based IPS algorithm showed a systematic delay in IC detection of 9.2 ms (LoA: 4.1 to 14.3 ms) and a −3.5 percentage point bias in ST pre-activity (LoA: −8.9 to 1.9% of MVC). In contrast, the criteria-based IPS algorithm, demonstrated minimal bias in IC detection (−0.1 ms; LoA: −3.5 to 3.4 ms) and ST pre-activity (−0.1% MVC; LoA: −1.9 to 1.7% of MVC). These findings suggest the criteria-based IPS algorithm enables accurate IC detection, supporting its potential for practical monitoring of ST pre-activity during sports-specific side-cutting outside laboratory environments.

## 1. Introduction

Non-contact anterior cruciate ligament (ACL) injuries in team sports predominantly occur within the first 50–70 ms after initial contact (IC) during side-cutting maneuvers and single-leg landings [1,2,3]. This short time window limits the contribution of the protective sensory feedback mechanisms. Consequently, hamstring muscle pre-activity prior to IC plays a critical role in stabilizing the knee joint and reducing ACL stress [4]. The hamstring muscle group functions as an ACL agonist by counteracting anterior tibial shear forces [5]. Moreover, research suggests that increased medial hamstring muscle (m. semitendinosus, ST) pre-activity, enhances compression of the medial knee joint compartment, thereby reducing external knee abduction moments and internal tibial rotation, both known ACL-injury risk mechanisms during side-cutting [6,7,8,9]. Despite this, ACL injuries remain prevalent among female team sports athletes, particularly among adolescents [10]. Thus, expanding the evidence base for accurate assessment of ST pre-activity levels during high-risk movements, e.g., side-cutting, in female athletes is warranted, ultimately to detect “insufficient” ST pre-activity, but also to enable testing under realistic, sport-specific conditions. Accordingly, there is a need for tools improving clinical practitioners’ ability to evaluate the in-field neuromuscular injury risk in female athletes during movements, such as side-cutting. Such tools may help to identify athletes with insufficient ST pre-activity who may be predisposed to future ACL injury [11,12,13], inform and evaluate rehabilitation practice [14,15] or injury prevention programs [16].

Recent advances in small wireless surface electromyography (sEMG) sensors have improved the feasibility of neuromuscular assessments in applied sports settings. However, accurate IC detection remains essential for quantifying ST pre-activity during high-risk movements. Even small deviations of a few milliseconds can substantially affect sEMG amplitudes calculated within the narrow 50 ms window prior to IC [6,11]. For this reason, assessment of ST pre-activity has traditionally been conducted in laboratory settings, where IC is determined from vertical ground reaction force (vGRF) data [6,11]. With the rapid development of wearable technologies [17,18], several studies have explored the use of inertial measurement units (IMUs) for in-field IC detecting during running, e.g., [19,20,21,22]. Whereas few studies have evaluated IMUs’ IC detection accuracy in high-dynamic sports movements [21,23,24], such as side-cutting, where most ACL injuries occur [1,2,3]. In addition to the added signal complexity of side-cutting compared to straight-line running, changes in running speeds [20,21,22], and even minor changes in IMU placement [25] have shown to affect IC timing during running. This sensitivity may limit the robustness of IMU-based IC detection in sport-specific side-cutting tasks within applied sports settings.

Insole pressure sensors (IPS), which have been available for decades [18,26], are another potential tool for in-field IC detection. IPS represent the second most commonly used wearable system for gait event detection after IMUs [18], and even served as the ground truth in validation studies [18]. IPS measure the pressure distribution between the foot and the sole from sensing technologies (e.g., optoelectronic or piezoelectric), combining pressure data from sensing elements. Numerous studies have evaluated the use of IPS for IC detection during straight-line running at different intensities, e.g., [27,28,29]. Blades et al. [29] demonstrated that different IC detection algorithms, ranging from simple threshold-crossing approaches to slope or first-derivative-based methods, identified IC with a mean absolute error between 0.7 ± 0.3 ms and 5.7 ± 0.8 ms during level treadmill running at speeds from 2.6 to 3.8 m/s. Similarly, Mann et al. [28] showed that their IPS-based system identified IC with a mean bias close to zero and limits of agreement (LoA) below 3 ms at self-selected treadmill running speeds (3.0 ± 1.0 m/s). Despite this promising potential, researchers have paid limited attention to IPS as a tool for in-field IC detection during side-cutting maneuvers in sports settings [29,30].

To our knowledge, no study has examined how different IPS-based IC detection methods influence the assessment of ST pre-activity during side-cutting. Therefore, this study aimed to (a) develop IPS-based algorithms to detect IC during anticipated sport-specific side-cutting, (b) evaluate agreement between IPS-derived IC and gold-standard vGRF measurements, and (c) determine agreement in ST pre-activity when IC was identified using IPS algorithms versus vGRF data.

## 2. Materials and Methods

### 2.1. Participants

Twenty-six physically active and injury-free female athletes, two handball and 24 football players (age: 26.1 ± 4.0 years; height: 167.0 ± 4.8 cm; body mass: 65.9 ± 8.9 kg) volunteered to participate in this study. Participants were excluded if they were younger than 18 years or had any current injury, illness, or other conditions that limited full participation in regular training or match play at the time of testing. Prior to enrollment, all participants were informed of the purpose of the study and procedures. Written informed consent was obtained from all participants in accordance with the Declaration of Helsinki. The study protocol was assessed by the local ethics committee in the Capital Region of Denmark and was deemed not to require a full ethical review (F-23047710).

### 2.2. Data Collection and Processing

Following a standardized warm-up protocol consisting of running, jumping, and squatting exercises with progressively increasing intensity from submaximal to maximal effort, participants performed three isometric maximal voluntary contractions (MVCs) of the hamstring muscles in a prone position with the knee flexed to 10° [11]. Subsequently, after a minimum of five familiarization trials, participants performed an individualized number of anticipated side-cutting maneuvers on their preferred pivoting leg until five successful trials were obtained (i.e., the entire pivot foot placed on the force plate). The preferred pivoting leg was defined as the leg opposite the preferred kicking leg for football players and the leg contralateral to the dominant throwing arm for handball players. Participants were instructed to perform the side-cut at maximal effort, simulating game conditions [11]. Neither approach speed nor cutting angle was standardized, allowing participants to execute their individual sport-specific side-cutting maneuver.

The vGRF signals were recorded at 1000 Hz for the pivoting step using a floor-embedded AMTI force plate (BMS400600, Advanced Mechanical Technology Inc., Watertown, MA, USA). Vertical ground reaction forces, IPS, and sEMG data were synchronized and collected using myoResearch software (MR 3.17, Noraxon Inc., Scottsdale, AZ, USA). Subsequent signal processing was performed in MATLAB (v.23.2, MathWorks^®^, Natick, MA, USA). The vGRF-based IC was defined as the instant where the signal exceeded a threshold of 20 N [13].

#### 2.2.1. Insole Pressure Sensors Measurements

IPS data were recorded at 1000 Hz using SmartLead Ultium^®^ insoles (insole thickness: 3.5 mm; measurement range: 0–75 psi [0–51.7 N/cm^2^]). The insoles were placed inside the participants’ shoes, on top of the existing shoe sole, using the appropriate individual insole size (S, M, or L) and calibrated to the participant’s body weight (BW) prior to testing. Pressure data were obtained from four zones: (1) heel (combined medial and lateral heel sensors), (2) lateral longitudinal arch (arch sensor), (3) metatarsals (combined first, third, and fifth metatarsal sensors), and (4) hallux/toes (combined hallux and toe sensors). A single pressure value was generated from the sum of the four sensor zones and used for subsequent IC detection analyses.

#### 2.2.2. ST sEMG Measurements

ST sEMG of the participants’ pivoting leg were recorded during side-cutting and hamstring maximal voluntary contraction (MVC) trials using wireless SmartLead Ultium^®^ sEMG sensors (Ultium^®^ EMG, Noraxon Inc., Scottsdale, AZ, USA) sampling at 4000 Hz. Electrode placement over the ST was determined by palpation with the participant standing, the knee flexed, and the hip and knee slightly internally rotated to preferentially activate the medial hamstring muscles. Dual surface electrodes with a 2.0 cm inter-electrode distance (Ambu^®^ Blue Sensor N ECG, N-00-S/25, Ambu, Ballerup, Denmark) were positioned over the most prominent portion of the ST muscle belly and aligned parallel to the muscle fiber direction. Prior to electrode placement, the skin was shaved, lightly abraded with fine sandpaper, and cleansed with an alcohol wipe to reduce impedance. No adverse reactions were observed following this procedure. Skin impedance and signal noise were considered acceptable if they were below 10 kΩ and 2 µV, respectively. Moreover, sEMG electrodes were fixed with adhesive tape and an elastic compression garment to reduce movement artifacts.

Following data collection, sEMG signals were high-pass filtered using a fourth-order Butterworth filter with a 20 Hz cutoff frequency and subsequently smoothed using a root mean square (RMS) algorithm with a 30 ms moving window, as previously described [6,11]. The processed sEMG signals obtained during side-cutting were normalized to the maximal amplitude of the filtered sEMG recorded during the three hamstring MVC trials. Finally, ST pre-activity was calculated from the processed side-cutting sEMG as the mean activity during the 50 ms preceding IC [6,11].

### 2.3. IPS IC Detection Development

**Figure 1:**
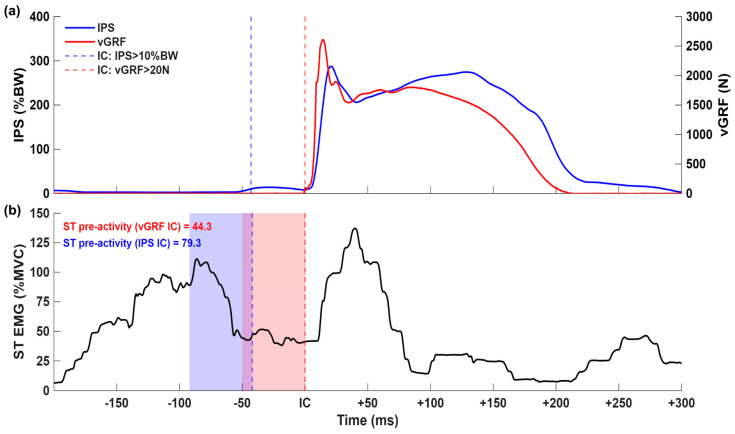
Representative side-cutting trial. (**a**) IPS and vGRF data, along with initial contact (IC) identified using an IPS-based threshold (>10%BW, blue dotted line) and a vGRF-based threshold (>20 N, red dotted line). (**b**) Normalized ST sEMG activity. Shaded regions represent the 50 ms pre-activity period preceding IC, determined from vGRF (red) and IPS (blue), respectively. Note the early IC detection from the IPS-derived IC due to in-shoe pressure, and its effect on ST pre-activity levels.

Initially, a simple 10%BW threshold crossing approach was used to determine IC from the IPS data. The use of an absolute threshold resulted in early IC detection in multiple trials due to non-zero pressure values in the swing phase prior to IC (Figure 1), an issue previously reported in sprinting studies [27]. Thus, more advanced approaches were considered.

#### 2.3.1. IPS Relative BW Threshold Crossing Algorithm

**Figure 2:**
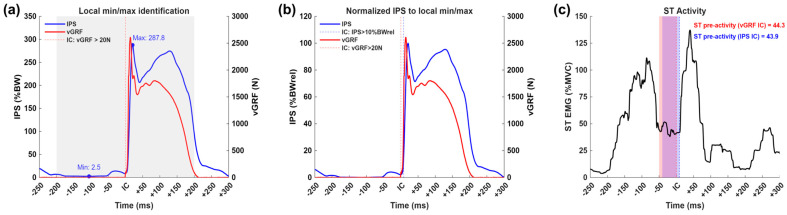
(**a**) IPS and vGRF data from a representative side-cutting trial, and the identification of local minimum and maximum IPS values within a span of 200 ms before and after vGRF-based IC (dotted red line). (**b**) IPS normalized to the identified local minimum and maximum values, and IC detected using the BWrel algorithm (>10%BWrel, dotted blue line). (**c**) ST pre-activity sEMG determined using IC defined by vGRF and BWrel algorithms, respectively.

To overcome the issue of non-zeroing pressure values in the preceding flight-phase, a BW relative (BWrel) threshold crossing algorithm was developed, where the original IPS data was normalized to local minimum and maximum values obtained from a search window 200 ms prior to 200 ms post the vGRF-based IC for each trial (Figure 2). Subsequently, IC was defined as the instant when BWrel pressure data exceeded a 10%BWrel threshold.

#### 2.3.2. IPS Criteria-Based Algorithm

**Figure 3:**
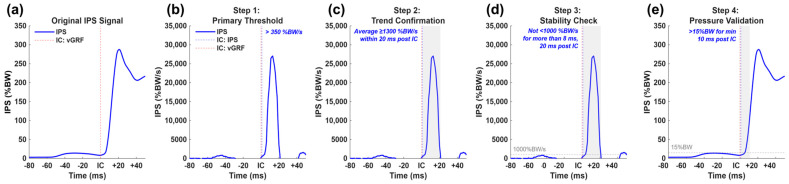
(**a**) Original IPS signal and vGRF-derived IC (red dotted line). (**b**) First derivative of the IPS signal, with IC detected using a 350%BW/s threshold crossing (dotted blue line), which defines the primary threshold in the criteria-based IPS algorithm. (**c**) The IC robustness step. (**d**) Trend confirmation step. (**d**) Stability check step. (**e**) Pressure validation step.

Additionally, we developed a criteria-based algorithm to identify IC from IPS data. We designed the algorithm through visual inspection of side-cutting pressure signals from 22 participants (≈85% of the sample) and tuned the threshold values for the derivative-based criteria using a systematic grid-search procedure, in which candidate parameter combinations were evaluated to minimize the error in IC timing relative to vGRF-based IC detection. The grid search was restricted to the three derivative-based thresholds, whereas the pressure validation criterion was not subject to systematic optimization. This process yielded four explicit criteria (Figure 3). The final algorithm was then applied to the full dataset. The algorithm confirmed IC only when all criteria were satisfied:

1. Primary threshold criteria: The first point at which the first derivative of the original IPS signal exceeded 350%BW/s.
2. Trend confirmation: 20 ms after the primary threshold is exceeded, the average of the IPS derived signal must exceed 1300%BW/s.
3. Stability check: 20 ms after the primary threshold is exceeded, the derived IPS signal must not fall below 1000%BW/s for more than 8 consecutive milliseconds.
4. Pressure validation: 10 ms after the primary threshold is exceeded, the IPS signal must be above 15%BW for at least 10 consecutive milliseconds.

The primary threshold (350%BW/s) detected the rapid increase in the first derivative of the IPS signal that characterizes the onset of IC. However, transient signal fluctuations could occasionally exceed this threshold without representing true contact events (non-zeroing flight-phase events). We therefore implemented three additional criteria to improve IC robustness identification.

The trend confirmation criterion ensured that the first derivative of the IPS signal rise continued in the subsequent 20 ms following the primary IC detection, as one would expect from a true foot-ground contact. The stability check criterion excluded short-duration threshold crossing, e.g., from in-flight toe pressure, within the 20 ms search window. Finally, the pressure validation criterion served as a second verification that the IPS signal reached and maintained a plausible pressure for the initial period after IC detection.

### 2.4. Statistics

Agreement between IC detection from IPS data, i.e., IPS BWrel threshold and criteria-based IPS algorithms, respectively, and the ground truth IC determined from vGRF data was evaluated using Bland–Altman analysis [31]. This analysis is used to quantify the mean difference (bias) and the 95% limits of agreement (LoA), defined as the mean difference ±1.96 SD, between IC detection methods. Similarly, Bland–Altman analysis was used to assess agreement in ST pre-activity obtained from the two IPS IC detection algorithms (IPS BWrel threshold crossing and criteria-based IPS algorithm, respectively), compared with ST pre-activity obtained from the vGRF IC detection method. Bland–Altman plots were constructed to show the mean value of IPS IC algorithms and vGRF threshold approach on the *x*-axis and the difference between IPS and vGRF approaches on the *y*-axis.

## 3. Results

### 3.1. IC Detection from IPS Data

**Figure 4:**
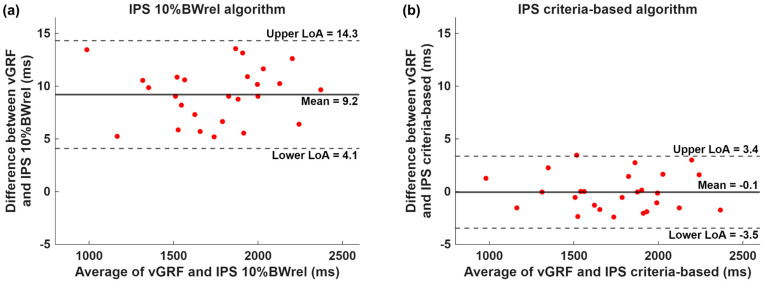
Bland–Altman plots illustrating agreement in IC detection between IPS-based methods and vGRF. panel. (**a**) Shows the IPS 10%BWrel threshold algorithm and panel. (**b**) Shows the IPS criteria-based algorithm. The solid black horizontal line represents the mean difference (bias), and the dashed black horizontal lines represent the upper and lower 95% limits of agreement, defined as the mean difference ±1.96 standard deviations.

Bland–Altman plots comparing the two IPS algorithms for IC detection are presented in Figure 4. For the IPS 10%BWrel algorithm, a systematic delay in IC detection was observed, with a mean bias of 9.2 ms and limits of agreement (LoA) ranging from 4.1 to 14.3 ms.

For the IPS criteria-based IC algorithm, IC was detected in all trials with all four criteria met. The Bland–Altman plot for the IPS criteria-based algorithm demonstrated a mean bias of −0.1 ms in IC detection (tendency to earlier IC detection), with LoA ranging from −3.5 to 3.4 ms.

### 3.2. ST Pre-Activity

**Figure 5:**
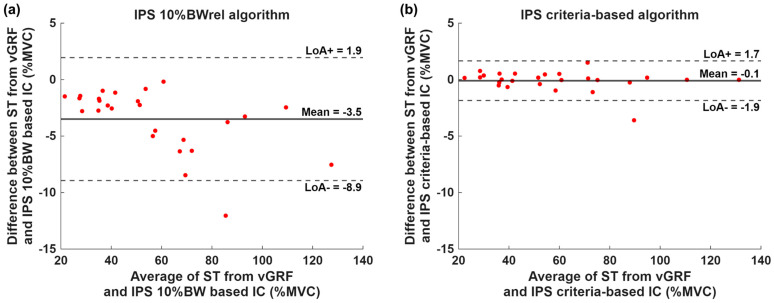
Bland–Altman plots display ST pre-activity when IC is determined from 10%BWrel algorithm (**a**) and criteria-based IPS algorithm (**b**), compared to IC identified from vGRF. Black horizontal lines display mean offset, and upper and lower limits of agreement (±1.96 standard deviation) are displayed as dashed horizontal black lines.

Average ST pre-activity was 58.5 ± 27.6% of maximal MVC when IC was determined using the ground-truth vGRF data, compared with 55.0 ± 26.0% and 58.4 ± 27.4% of maximal MVC, when IC was derived using the IPS 10%BWrel and criteria-based algorithms, respectively. The Bland–Altman plots (Figure 5) revealed a mean bias of −3.5 percentage points of maximal MVC (LoA: −8.9 to 1.9% of maximal MVC) and −0.1 percentage points of maximal MVC (LoA: −1.9 to 1.7% of maximal MVC) when IC was determined using the IPS 10%BWrel algorithm and the IPS criteria-based algorithm, respectively.

## 4. Discussion

We developed two IPS-based algorithms to detect IC during sport-specific side-cutting and evaluated their agreement with vGRF-based IC detection, and the subsequent influence on ST pre-activity levels. The criteria-based IPS algorithm demonstrated minimal bias in both IC detection (Figure 4b) and ST pre-activity (Figure 5b), whereas the refined body weight threshold crossing algorithm delayed IC identification (Figure 4a) and underestimated ST pre-activity (Figure 5a). These findings highlight the strong potential of combining IPS and sEMG sensors as a practical field-based approach for monitoring ST pre-activity during side-cutting. This approach has the potential to facilitate scalable neuromuscular assessment in applied team sport settings, thereby advancing our understanding of ST pre-activity as a risk factor for non-contact ACL injury in athletes [6,11].

This study demonstrates that the two IPS-based algorithms can detect IC for the pivoting step of side-cutting with accuracy comparable to that previously reported for straight-line running using IPS data [28,29]. Although methodological differences preclude direct comparison, the criteria-based IPS algorithm demonstrated comparatively small mean bias and narrow limits of agreement relative to the combined pelvis- and foot-mounted IMU-based approach proposed by Di Paolo et al. [23] for high-dynamic sports movements, including side-cutting (mean bias: −8.6 ms; LoA: −227.3 to 210.0 ms). In particular, the results from the criteria-based algorithm, which primarily utilize the first derivative of the pressure signal, were promising. Blades et al. [29] previously proposed the use of a threshold crossing in the first derivative of the IPS signal, rather than the absolute or refined pressure signal, for IC detection in running, an approach that is supported by our results. The three subsequent criteria (trend confirmation, stability check, and pressure validation) in our criteria-based IPS algorithm were primarily introduced to verify that the IPS signal reached and maintained a plausible increase in pressure during the initial contact phase, and to reduce false-positive IC detections arising from the 350%BW/s threshold crossing.

The 10%BWrel algorithm improved IC detection compared to a simple pressure-threshold crossing approach (Figure 1) but introduced a systematic 9.2 ms delay (LoA: 4.1–14.3 ms, Figure 4). Although IMU-based methods have shown a similar accuracy range in IC detection [23], this delay propagated to ST pre-activity levels in the present study, producing wide limits of agreement (–8.9 to 1.9% of maximal MVC; Figure 5). This is critical, as a 10 percentage point increase in ST pre-activity during side-cutting is reported to reduce ACL injury risk by 38% in female team sports athletes [11]. The delayed IC likely underlies the lower ST pre-activity observed, since hamstrings are less active during the initial contact phase when eccentric quadriceps activation is needed to counteract large knee flexion moments [6].

The high agreement observed when combining our criteria-based IPS algorithm and sEMG sensors for IC detection and ST pre-activity assessment highlights the strong potential for practical in-field neuromuscular evaluation in applied team sport settings. Assessing lower-limb muscle activity during dynamic tasks, particularly hamstring and quadriceps muscle activity, is critical given their agonistic and antagonistic roles in ACL loading [5]. This seems especially relevant for ACL injury prevention in female team sports, where reduced ST pre-activity during side-cutting has been identified as a risk factor for non-contact ACL injury [6,11].

The present IPS-based IC detection algorithms have several limitations that restrict their application in applied field settings. First, IC derived from vGRF was used to define the IC search window for the two IPS algorithms in this study, thereby limiting the generalizability of the proposed IC methods in applied team sports settings. Future approaches may address this limitation by identifying search windows for IC detection based on the timing of peak pressure or the first derivative peak during the pivot step. Stance time may serve as a criterion to distinguish the pivoting step, given that it is typically longer than both the preceding decelerations and subsequent reacceleration steps in side-cutting maneuvers [32]. However, further research is required to evaluate the accuracy of such approaches. Additionally, the IPS algorithms were specifically developed for change-of-direction tasks; thus, their generalizability to other high-dynamic movements, such as jump-landing activities, decelerations, or sprinting tasks, remains unknown. Previous work has shown that ST pre-activity during both single- and multiplanar jump tests does not replicate the neuromuscular ST demands of sport-specific side-cutting in female team sports athletes [13], suggesting that neuromuscular assessment may be most effective during side-cutting tasks. Similarly, the robustness of the criteria-based algorithm should be tested in male athletes and in athletes from other sports involving sport-specific side-cutting tasks to improve its generalizability across sports, gender, and age. Finally, we developed the criteria-based algorithm using 85% of the dataset on which it was subsequently validated, which may have introduced a degree of overfitting when applied to the full sample. Future studies should therefore validate the algorithm in an independent cohort to determine its generalizability in applied sports settings. Nevertheless, if these limitations are addressed and the criteria-based algorithm proves reliable, the proposed combination of IPS and sEMG data could potentially serve as a valuable tool for neuromuscular ACL injury assessment during side-cutting in applied team sports settings.

From an implementation perspective, future research should prioritize the development of low-tech, user-friendly processing pipelines that maintain measurement accuracy. For instance, to enhance the practicality and cost-effectiveness of in-field neuromuscular assessments, future work should investigate the feasibility of relying solely on integrated IMUs, commonly embedded in modern sEMG sensors, for IC detection, thus eliminating the need for separate systems. Overall, such initiatives may facilitate the broader application of neuromuscular assessments in applied team sport settings, ultimately expanding the evidence base for neuromuscular assessments in an ACL injury context.

## 5. Conclusions

The criteria-based IPS algorithm accurately detected IC during side-cutting and reduced systematic bias in IC detection, thereby improving the sEMG measures of m. semitendinosus pre-activity. This approach provides a potential in-field method to quantify hamstring pre-activity during side-cutting in team sports athletes and may support neuromuscular assessments outside laboratory settings in the context of ACL-injury risk assessment. In contrast, practitioners should interpret hamstring pre-activity levels with caution when IC is determined using body weight threshold crossings in the IPS signal, or other wearable sensor approaches (e.g., IMUs) with similar IC detection accuracies (±10 ms). Such methods may under- or overestimate athletes’ ST pre-activity levels during side-cutting and thereby potentially compromise clinical interpretation.

## Data Availability

The data supporting the findings of this study are available on reasonable requests from the corresponding author. The data is not publicly available due to privacy restrictions.

## Abbreviations

The following abbreviations are used in this manuscript:

ACL	Anterior Cruciate Ligament
BW	Body Weight
BWrel	Relative Body Weight
IC	Initial Contact
IMUs	inertial measurement units
IPS	Insole pressure sensor
LoA	Limits of Agreement
MVC	Maximum Voluntary Contraction
sEMG	Surface electromyography
ST	m. semitendinosus
vGRF	Vertical Ground Reaction Force
